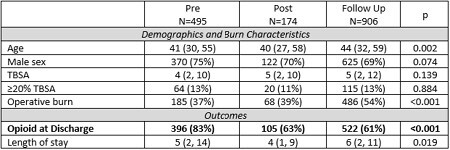# 726 Impact of Opioid-Minimizing Pain Protocols After Burn Injury: An Update of Previous Quality Improvement Project

**DOI:** 10.1093/jbcr/irae036.269

**Published:** 2024-04-17

**Authors:** Andrea Abeln, James Klugh, Deepanjli Donthula, Catherine Almand, Taylor Campbell, Chuantao Jiang, Daniel J Freet, Todd F Huzar, David J Wainwright, Charles E Wade, Lillian S Kao, John A Harvin

**Affiliations:** McGovern Medical School at UTHealth Houston, Houston, TX; McGovern Medical School at UTHealth Houston, Houston, TX; McGovern Medical School at UTHealth Houston, Houston, TX; McGovern Medical School at UTHealth Houston, Houston, TX; McGovern Medical School at UTHealth Houston, Houston, TX; McGovern Medical School at UTHealth Houston, Houston, TX; McGovern Medical School at UTHealth Houston, Houston, TX; McGovern Medical School at UTHealth Houston, Houston, TX; McGovern Medical School at UTHealth Houston, Houston, TX; McGovern Medical School at UTHealth Houston, Houston, TX; McGovern Medical School at UTHealth Houston, Houston, TX; McGovern Medical School at UTHealth Houston, Houston, TX

## Abstract

**Introduction:**

In 2019, a single-burn center QI project resulted in a significant reduction in opioid prescribing at discharge. Such reductions were achieved through implementation of a pill-based, opioid-minimizing pain protocol. After completion, no additional QI interventions were sustained other than dissemination of the protocols to providers on the unit. We hypothesized that the reductions in opioid prescribing at discharge have been sustained since the QI project ended.

**Methods:**

Three groups of patients admitted to the burn service were compared: 1) Pre (01/2018 to 07/2019), 2) Post (01/2020 to 06/2020), and 3) Follow up (7/2020 to 3/2023). The Pre and Post groups were the same as described in the original QI report. The protocol was implemented from 08/2019 to 12/2019 and these patients are not included. Patient demographics, burn characteristics, and lengths of stay were abstracted from the burn registry. Opioid prescriptions at discharge were obtained from the electronic medical record. The primary outcome was opioid prescribing at discharge. Groups were compared using Kruskal-Wallis rank test, Pearson’s Chi-squared, and Fisher’s Exact test for continuous, binary, and sparse binary outcomes, respectively.

**Results:**

A total of 1,575 patients were included: 495 Pre Group, 174 Post Group, and 906 Follow Up Group. Patients differed between cohorts in median age and proportion of operative burns. The Follow Up Group had a continued reduction in opioid prescribing at discharge (61%) as compared to the Pre Group (83%). The most prescribed opioids at discharge in the Follow Up Group were: 1) oxycodone (76%), 2) tramadol (21%), and hydrocodone (3%).

**Conclusions:**

The previously found reduction in opioid prescribing at discharge after implementation of opioid-minimizing protocols for acute burn pain were found to be sustained. This finding was present despite the significant increase in operative burns admitted to the burn center.

**Applicability of Research to Practice:**

Long-term reduction of opioid prescribing at discharge can be achieved using opioid-minimizing acute pain protocols.